# Inhibition of Several Bacterial Species Isolated from Squid and Shrimp Skewers by Different Natural Edible Compounds

**DOI:** 10.3390/foods11050757

**Published:** 2022-03-06

**Authors:** Lourenço Pinto de Rezende, Joana Bastos Barbosa, Ana Maria Gomes, Ana Machado Silva, Daniela Fonseca Correia, Paula Teixeira

**Affiliations:** 1 Laboratório Associado, Escola Superior de Biotecnologia, CBQF—Centro de Biotecnologia e Química Fina, Universidade Católica Portuguesa, 4169-005 Porto, Portugal; pinto.rezende88@gmail.com (L.P.d.R.); amgomes@ucp.pt (A.M.G.); pcteixeira@ucp.pt (P.T.); 2 SONAE, Lugar do Espido, Via Norte, 4471-909 Maia, Portugal; amsilva@sonaemc.com (A.M.S.); dafcorreia@sonaemc.com (D.F.C.)

**Keywords:** antimicrobial activity, Citrox^®^, seafood products, spoilage

## Abstract

Seafood is an excellent source of nutrients, essential for a healthy diet, ranging from proteins and fatty acids to vitamins and minerals. Seafood products are highly perishable foods due to their nutritional characteristics and composition. The application of nontoxic, natural, and edible preservatives to extend the shelf-life and inhibit bacterial proliferation of several foods has been a hot topic. Consequently, this work aimed to perform the microbiological characterization of squid and shrimp skewers during their shelf-life (five days) and evaluate the susceptibility of randomly isolated microorganisms to several natural edible compounds so that their application for the preservation and shelf-life extension of the product might be analyzed in the future. The product had considerably high total microorganisms loads of about 5 log CFU/g at day zero and 9 log CFU/g at day five. In addition, high bacterial counts of *Pseudomonas* spp., Enterobacterales, and lactic acid bacteria (LAB) were found, especially on the last day of storage, being *Pseudomonas* the dominant genus. However, no *Escherichia coli* or *Listeria monocytogenes* were detected on the analyzed samples. One hundred bacterial isolates were randomly selected and identified through 16s rRNA sequencing, resulting in the detection of several Enterobacterales, *Pseudomonas* spp., and LAB. The antibacterial activity of carvacrol, olive leaf extract, limonene, Citrox^®^, different chitosans, and ethanolic propolis extracts was evaluated by the agar diffusion method, and the minimum inhibitory concentration was determined only for Citrox^®^ since only this solution could inhibit all the identified isolates. At concentrations higher than or equal to 1.69% (*v/v*), Citrox^®^ demonstrated bacteriostatic and bactericidal activity to 97% and 3% of the isolates, respectively. To our knowledge, there are no available data about the effectiveness of this commercial product on seafood isolates. Although preliminary, this study showed evidence that Citrox^®^ has the potential to be used as a natural preservative in these seafood products, improving food safety and quality while reducing waste. However, further studies are required, such as developing a Citrox^®^-based coating and its application on this matrix to validate its antimicrobial effect.

## 1. Introduction

Seafood is regarded as a great source of nutrients, essential for a healthy diet, ranging from proteins and fatty acids to vitamins and minerals [1]. Due to this, there is an increasing demand for new, fresh, precooked, and ready-to-eat seafood products [2]. However, their nutritional characteristics and composition turn seafood products into highly perishable foods. The resulting degradation leads to large quantities of product waste [3], culminating in financial losses and quality concerns for both the industry and the consumers [1].

The degradation of fish products by the activity of specific spoilage organisms (SSOs) is the most concerning cause of spoilage of products faced by the producers and retailers [4]. Particular species of spoilage organisms can vary depending on the origin of the product and the processing techniques employed during its handling and storage [5]. In the case of seafood products, most of the spoiling agents are Gram-negative psychrotolerant bacteria. Of these, there is an over-representation of genera belonging to the phylum of Proteobacteria [6], such as *Pseudomonas*, *Vibrio*, *Moraxella*, *Shewanella*, *Acinetobacter*, *Aeromonas*, and *Photobacterium*. Gram-positive bacteria of the genera *Micrococcus* and *Clostridium*, as well as lactic acid bacteria (LAB), can also be present [6,7]. In shellfish, high concentrations of nonprotein nitrogen compounds and pH values typically above 6.0 represent excellent conditions for the proliferation of SSOs of the *Morganella*, *Proteus*, and *Pseudomonas* genera [7].

The production of chemical compounds such as trimethylamine, ammonia, hydrogen sulphide, methylmercaptan, and dimethyl-disulphide from the reduction of trimethylamine oxide, the metabolization of urea or deamination of amino acids, and the breakdown of sulphurous compounds are typical consequences of the proliferation of bacteria such as *Shewanella* spp., LAB, and *Photobacterium* spp. [6]. Additionally, the production of biogenic amines, such as cadaverine, putrescine, and histamine, can be induced by the decarboxylation of amino acids by bacteria such as *Shewanella putrefaciens, Hafnia alvei*, and *Morganella morganii* [6].

The growth of pathogenic microorganisms in food products also demands great attention. Seafood-associated illnesses have been linked to a variety of viruses, bacteria, and parasites [8]. The presence of *Vibrio* spp., *Clostridium* spp., *Salmonella* spp., *Shigella* spp., and some strains of *Staphylococcus aureus* can induce severe disease and is of paramount importance when evaluating whether a product is safe for consumption or not. These pathogens are primarily present in food products due to the contamination and pollution of the environments from which the product originated [8]. Moreover, contamination of food products with pathogens, resulting from the cross-contamination of products and/or from the migration of microorganisms from the gastrointestinal tracts, such as *Listeria monocytogenes* [9] and some strains of *Escherichia coli*, is typically the result of poor handling practices in food processing [10,11]. This intestine to muscle migration can occur if the period between death and evisceration of the animal is too long [9]. In addition to resulting in loss of nutritional value, the presence of pathogens in food products can also lead to serious foodborne illnesses [12].

Due to increasing consumer demands to reduce the use of synthetic chemicals in food, intense pressure to innovate and to provide natural alternatives has been felt all over the food industry [13]. The application of nontoxic, edible preservatives to extend shelf-life and inhibit bacterial proliferation in seafood has been the focus of several recent studies [14,15,16]. These preservatives have the potential to retard the spoilage of the product, retaining the hedonic characteristics of seafood, such as smell, texture, and flavor [16]. Natural antimicrobials, such as limonene, thymol, oleuropein, carvacrol, propolis, and chitosan, present valuable alternatives to the undesirable synthetic antimicrobials [15,16,17,18,19,20]. The nonsynthetic nature of these compounds provides the treated products with an all-natural characteristic, contributing to a more significant appeasement of consumer interests. For a product to be accepted as a potential additive to food products, every component should be food-grade. Thus, no safety issue should arise when using the product under the recommended conditions. On account of the inherent safety of food-grade products, natural and edible solutions can be applied to most food products to extend their shelf-life [21]. As a consequence of their popularity as promising innovations, numerous components demonstrating antimicrobial activity have been incorporated into these edible solutions with positive results [15,16,22].

This work focused on analyzing the antibacterial activity of several natural compounds against the proliferation of bacteria isolated from uncooked seafood products consisting of squid (*Loligo duvauceli*) and shrimp (*Parapenaeopsis*, *Penaeus*, and *Metapenaeus* genera). Being this an extremely perishable food product, the inhibition of bacteria collected from the product with nonsynthetic, natural, and edible compounds can open the way to the formulation of efficient and safe coatings and treatments, capable of extending the shelf-life of such perishable products while maintaining an all-natural label.

## 2. Materials and Methods

### 2.1. Microbiological Characterisation of Squids and Shrimps Skewers

The product (two units of each of two lots) was transported to the laboratory under refrigeration conditions and analyzed immediately and after 2 and 5 days of refrigerated storage (stored at 4 °C and at not controlled atmospheric conditions in the original plastic boxes). Due to a shelf-life of 3 to 4 days, such periods were selected to represent a fresh product, one halfway through its shelf-life limit, and another after its shelf-life. This shelf-life period is a result of the sensorial perception of degradation observed during storage, being the product’s organoleptic characteristics unfit for consumption after 4 days of refrigerated storage.

To achieve reproducibility and representative samples, two units of each lot were mixed to perform a composite sample. Each sample was composed of 10 g of squid mantle, 5 g of squid tentacles, and 10 g of shrimp meat (with no exoskeleton). According to standard microbiological methods, each of these 25 g samples were added to 225 mL of sterile buffered peptone water (Biokar Diagnostics, Beauvais, France) and homogenized in a stomacher (Interscience, Saint Nom la Brèteche, France) for 2 min. Appropriate decimal dilutions were prepared in Ringer’s solution (Biokar Diagnostics) for microbial enumeration: colony counts of viable microorganisms at 30 °C for 72 h on Plate Count Agar (PCA, Biokar Diagnostics; ISO 4833-1 [23]); LAB at 30 °C for 72 h on de Man-Rogosa and Sharpe agar (MRSA, Biokar Diagnosis; ISO 15214 [24]); *Pseudomonas* spp. counts at 30 °C for 48 h on *Pseudomonas* Agar Base (PAB, Biokar Diagnostics, ISO 13720 [25]) supplemented with cetrimide, fusidic acid and cefaloridin (VWR, Alfragide, Portugal); Enterobacterales at 37 °C for 24 h on RAPID′ Enterobacteriaceae medium (Bio-Rad, Hercules, CA, USA; ISO 21528 [26]); *Listeria monocytogenes* at 37 °C for 24 h on Agar *Listeria* Ottaviani and Agosti (ALOA, Biokar Diagnosis, ISO 11290-2 [27]); *Escherichia coli* at 44 °C for 24 h on Tryptone Bile X-Glucuronide Medium (TBX, bioMérieux; ISO 16649-1 [28]). Since the recommended storage of the product is at refrigeration temperatures, bacterial enumeration was also performed in all previously mentioned growth culture media at 11 °C.

### 2.2. Selection of Isolates

After enumeration, about 10% of colonies from each countable dilution plate were randomly selected. Presumptively, only presumptive colonies of *Pseudomonas* spp. and LAB were confirmed since the other microorganisms were grown on chromogenic (RAPID′ *Enterobacteriaceae* and TBX) or nonselective (PCA) culture media. Characteristic colonies in terms of color, type, elevation, and opacity were confirmed by Gram staining and oxidase test (*Pseudomonas* spp.) and Gram staining and catalase test (LAB).

Each randomly selected colony was purified by repeated streaking onto the general growth medium TSAYE—Trypticase Soy Agar (Biokar Diagnostics) with 0.6% Yeast Extract (Biokar Diagnostics) or MRSA (in the case of LAB) and incubated for 24 to 72 h under the same conditions. All isolates (10%) recovered were stored at −80 °C in Tryptic Soy Broth (TSB, Pronadisa) or MRS broth (only LAB) with 30% (*v/v*) of glycerol (Sigma, Steinheim, Germany) and subcultured twice before use.

### 2.3. Identification of Selected Isolates by 16S rRNA Sequencing

Each selected isolate was identified by 16S rRNA sequencing. The DNA was extracted according to the protocols for total DNA purification from Gram-positive or -negative bacteria of the GRS genomic DNA Kit (Grisp, Porto, Portugal). The primers used for the amplification of the gene fragments for the 16s rRNA identification were 27F (5′-AGAGTTTGATCCTGGCTCAG-3′) and 1492R (5′-GGTTACCTTGTTACGACTT-3′) [29]. For the preparation of the master mix, the following solutions were used: dNTP (10 mM), Taq buffer KCl (10×), MgCl2 (25 mM), primers 27F and 1492R (100 μM each), Taq polymerase (1 U/mL), and 2 μL of bacterial DNA. Amplification conditions were performed in a Thermocycler (Bio-Rad) following the above-mentioned protocol: one cycle at 94 °C for 5.5 min, a first phase encompassing both start and denaturation cycles; 30 alternating cycles of annealing and extension for 30 s at 55 °C and 90 s at 72 °C, respectively; one final cycle at 72 °C for 90 s for the final extension. After the last cycle, the products were cooled to 12 °C. PCR product purification was performed according to the GRS PCR and Gel Purification Kit (Grisp) protocol and used as templates. The resulting DNA sequences were aligned and compared in the Gene Bank through the use of the BLAST program (http://www.ncbi.nlm.nih.gov, accessed on 20 June 2020) [30].

### 2.4. Study of Antimicrobial Activity of Several Natural Products

The antimicrobial activity of ten natural products was tested against each selected isolate, and the most effective natural compound was chosen for further experiments.

#### 2.4.1. Preparation of the Inoculum

Inoculation of each isolate was performed from each culture grown on TSAYE or MRS (Biokar Diagnosis) at 30 °C for 24 h, by suspending isolated colonies in Ringer’s solution to obtain a turbidity equivalent to 0.5 McFarland scale.

#### 2.4.2. Preparation of Natural Products

##### Ethanolic Propolis Extracts (EPE)

Two propolis samples from different geographic locations were used: one sample collected from Maia, Porto, Portugal (EPE1) and the other from Vila Franca, Viana do Castelo, Portugal (EPE2). Stock solutions of 0.1 g/mL ethanolic propolis extracts were prepared for each sample, according to Casquete et al. [17]. Each propolis sample was extracted in 95% (*v/v*) ethanol (Sigma) for 24 h with magnetic agitation. Afterwards, the resulting mixture was filtered, and the residue was re-extracted in the same conditions. The filtrate solutions were combined, and each extract (0.1 g/mL) was stored at room temperature in the dark.

##### Chitosan Solutions

Chitosans of different molecular weights were obtained from Sigma-Aldrich (St. Louis, USA): low molecular weight (LMW; deacetylation degree of 75–85%), medium molecular weight (MMW; deacetylation degree of 75–85%), and high molecular weight (HMW1; >75%). In addition, another chitosan of HMW was used (HMW2; Aqua Premier Co., Thailand; deacetylation degree of 90%). Chitosan solutions were prepared in a 3% (*v/v*) solution of glacial acetic acid 99% (Panreac, Barcelona, Spain) to a final concentration of 30 mg/mL, according to Casquete et al. [31]. Afterwards, the solution was stirred overnight for the dissolution of chitosan.

##### Carvacrol and Limonene Solutions

Solutions of 0.05% (*v/v*) carvacrol (99%, Sigma-Aldrich) and 0.05% (*v/v*) limonene (≥95%, Sigma-Aldrich) were prepared by dissolution in polysorbate 80 (Sigma-Aldrich). The solutions were prepared just before usage.

##### Olive Leaf Extract Solution

A solution of 1.5% (*w/v*) olive leaf extract (Nutexa, Hoylake, UK) was prepared by dissolution of 0.3 g of the extract in 20 mL sterile distilled water. Since the active compound oleuropein is presented in 20% (*w/w*) of the olive leaf extract, only 0.3% (*w/v*) of oleuropein was present in the final solution.

##### Citrox^®^ Solution

The Citrox^®^ solution used was Citrox—ProGarda™ Fruit and Vegetable Decontaminant (ref. 14WP; Cirras Ltd., Middlesbrough, England), and it was diluted in sterile distilled water. Citrox—ProGarda™ Fruit and Vegetable Decontaminant is a natural, food-grade processing aid, free of genetically modified organisms, based on a citrus extract, with a combination of phenolic compounds and organic acids as its active ingredients. Dilutions were prepared in sterile water, and a solution of 50% (*v/v*) was used in the antimicrobial assay.

#### 2.4.3. Antimicrobial Activity Screening of Each Natural Solution

The antimicrobial activity of each solution was evaluated by the agar diffusion method [32]. Each solution was combined with the emulsifier polysorbate 80 in the proportion of 1:10 (polysorbate 80: natural compound solution). Blank disks (Oxoid, Basingstoke, United Kingdom) were immersed in each solution and left submerged for 30 min to absorb the solution. Sterile swabs were immersed in each inoculum suspension (prepared as described in Section 2.4.1) and spread in Müeller-Hinton Agar (MHA, Biokar diagnostics) plates. Disks containing the solutions were added to the top of the culture medium, and plates were incubated at 30 °C for 24 h. Disks adsorbed with distilled water, polysorbate 80, acetic acid (PanReac), and ethanol (Sigma) were used as controls.

#### 2.4.4. Minimum Inhibitory and Bactericidal Concentrations of Effective Natural Compounds

Minimum inhibitory (MIC) and bactericidal (MBC) concentrations were assessed only for Citrox^®^ solution, which was the only product showing antimicrobial activity for the majority of the isolates. Following the protocol for microdilution broth susceptibility test described by Aumeeruddy-Elalfi et al. [33], 100 μL of a solution containing 10 μL of polysorbate 80 and 90 μL of Müeller-Hinton Broth was distributed to each well of the 96-well microplates. The Citrox^®^ solution was diluted, and eight different concentrations, ranging from 0.39% to 50% (*v/v*), were distributed throughout the wells. Then, 50 μL of each inoculum, with a turbidity equivalent to 0.5 McFarland scale, was added to each well, and the microplates were incubated at 30 °C for 24 h. After the incubation period, 40 μL of a 0.02% (*w/v*) solution of iodonitrotetrazolium (INT) chloride Biochema dye (PanReac) was added to each well. The reaction between INT chloride and viable bacterial cells in the suspension induced a change of color, from a transparent mild yellow to an intense red due to the reduction of INT chloride into INT formazan. The lowest concentration of Citrox^®^ in which there was no change of color was considered the MIC.

To determine whether the compound was bactericidal or bacteriostatic, 10 μL of suspension was collected from the wells not experiencing a color change in the microplate and inoculated on Müeller-Hinton Agar. The plates were incubated at 30 °C for 24 h and checked for the presence of growth.

## 3. Results

Microbiological enumeration was performed for samples of two batches immediately after reception (0 days) and after 2 and 5 days of storage, according to each specific ISO standard and at 11 °C. The obtained results (log CFU/g) are presented in Table 1.

Isolated colonies from samples enumerated immediately after the reception were identified through 16s rRNA sequencing. In Table 2 and Table 3 are shown the medium from which these isolates were recovered and the several species identified, respectively.

The results of the antimicrobial activity assay with the respective diameter of inhibition halos observed for each compound against each identified isolate are presented in Table 4.

Sensitivity was assumed when an inhibition halo equal or superior to 10 mm in diameter was observed (Table 4). The percent sensitivity was calculated by dividing the number of inhibited isolates by the number of isolates from each type (*Pseudomonas*, Enterobacterales, or LAB) or by the total number of isolates. These results are presented in Table 5.

Of all the products investigated, only Citrox^®^ inhibited all of the selected isolates (Table 4 and Table 5). For this reason, only minimum inhibitory concentrations of the Citrox^®^ solution were further determined. Concentrations ranging from 0.03% to 50% (*v/v*) Citrox^®^ solution were prepared with sterile deionized water, and minimum inhibitory concentrations were determined. The results obtained are shown in Table 6.

## 4. Discussion

### 4.1. Microbiological Characterisation of Squid and Shrimp Skewers

No *L. monocytogenes* or *E. coli* were detected in the analyzed products. Despite not being common, the presence of *L. monocytogenes* [34] and *E. coli* [10] in seafood may occur [35], and the contamination by these pathogens is usually associated with unsafe handling of food during processing [11]. The absence of *E. coli* and *L. monocytogenes* suggests that safety procedures may have been applied during production.

Enumeration performed immediately after reception of the product showed a high microbial load for all parameters tested. *Pseudomonas* spp. seemed to be the most abundant type of bacteria with over 6 log CFU/g at the moment of reception, followed by members of the Enterobacterales order, with over 4 log CFU/g, and LAB, with just under 4 log CFU/g. *Pseudomonas* spp. are among the most noteworthy spoiling organisms in seafood [36].

The storage of the product under refrigerated conditions resulted in a considerable growth of bacterial communities during the five days of testing, with an increase of 3 to 4 log CFU/g observed for all the microbiological parameters. This increase was mainly observed on the second day of storage. This contrasts with what was observed by Don et al. [37] and Farajzadeh et al. [38], where microbial levels of shrimp only surpassed 8 log CFU/g after 12–14 and 8–10 days, respectively. This can be explained by these studies focusing exclusively on shrimp and because some irregularity in bacterial counts is expected depending on the capture zone and processing facility. Moreover, the skewers analyzed in the present study are subject to considerably more human manipulation than the food matrices studied by Don et al. [37] and Farajzadeh et al. were [38].

### 4.2. Identification of Selected Isolates by 16S rRNA Sequencing

Of the 100 randomly selected isolates, 50 were identified as members of the Enterobacterales order (according to Adeolu et al. [39]), being these distributed by the Enterobacteriaceae (*Klebsiella* genus), Hafniaceae (*Hafnia* and *Obesumbacterium* genus), Yersiniaceae (*Serratia* and *Rahnella* genus), Morganellaceae (*Morganella* genus), and Erwiniaceae (*Pantoea* genus) families [39]. Bacteria of the *Serratia* genus (*Serratia liquefaciens, Serratia nematodiphila*, and *Serratia fonticola*), commonly found in the environment, such as in soil, water, and intestinal tract of digestive tracts of various animals, represented over 20% of all isolates identified in the studied product. The facultative anaerobic bacteria *S. liquefaciens*, after *Serratia marcescens*, is the most common *Serratia* species involved in human infections, being previously connected with non-foodborne infections. [40]. Fourteen isolates were identified as *Rahnella* spp., another genus of the Yersiniaceae family, that can be found in soil, water, and intestinal tracts of herbivores [41]. *Rahnella aquatilis* is a fish pathogen that induces hemorrhagic septicemias in vulnerable hosts. In humans, *R. aquatilis* is an opportunistic pathogen since it can cause various infections, from wound infection to sepsis [41]. Two isolates were also identified as *Klebsiella oxytoca*, which is recognized as a pathogen of interest, due to its opportunistic characteristics and resistance to antibiotics [42]. This bacterium is usually present in water and soil and can be found in the intestine tracts of several animals, including humans [42]. Its presence in ready-to-eat food products has been previously reported by Nyenje et al. [43], alerting to unsafe practices that can pose serious threats to consumers. *Hafnia alvei* and *Morganella morganii* were also detected in the samples analyzed (6 and 3 isolates, respectively), and, as reported by Montet and Ray [6], these organisms are responsible for the production of biogenic amines in food products.

None of the 24 isolates of *Pseudomonas* spp. found are known to be human pathogens, being all the species widespread in various environments [44]. However, *Pseudomonas* spp., and more specifically *Pseudomonas fragi* (two isolates identified as *P. fragi*), comprise one of the most relevant genera responsible for the spoilage of shrimp in cold storage conditions [7]. The presence of this microorganism and its production of enzymes leads to the decomposition of the product and the surge of unpleasant sensorial changes [36]. While none of the isolates of *Pseudomonas* were identified as *Pseudomonas aeruginosa*, the most significant opportunistic pathogen of this genus [36], the presence of several species of this genus indicates the existence of ideal growing conditions for the proliferation of such a spoiling-inducing genus in this product. Any shelf-life-extending technique or compound to be applied to this shrimp and squid skewer should strive for the inhibition or elimination of *Pseudomonas* growth and microbial load.

Regarding LAB, *Lactococcus* spp. and *Leuconostoc* spp. were found in high numbers. *Leuconostoc* spp. are mesophilic and psychrotolerant bacteria of the *Lactobacillaceae* family, and some species such as *Leuconostoc gelidum* are commonly found in meat and fish products [45]. Production of lactic and acetic acids by *Leuconostoc* spp. through the fermentation of carbohydrates is a cause of food spoilage. Formation of slime, discoloration, and alteration of smells and flavors are also products of *Leuconostoc* activity [46,47]. While negative effects on food products can ensue from the proliferation of these bacteria, some *Leuconostoc mesenteroides* and *Leuconostoc lactis* strains have been recorded as potentially probiotic and bacteriocins producers, having antibacterial activity against the foodborne pathogen *L. monocytogenes* [48,49,50]. *Lactococcus piscium* was previously identified as a fish pathogen in diseased rainbow trout [51]. However, the relation between the presence of this bacteria and the disease has never been established [52], unlike its relation to food spoilage. This spoilage is, however, dependent on the strain and the type of foodstuff; *Lac. piscium* is known to cause the spoilage of meat but seems to have no impact on the degradation of seafood [52]. *Lactococcus gerviae* has been shown to cause “septicemias, ophthalmia, and hemorrhages” in marine farmed fish. In humans, *Lac. gerviae* has been the cause of some infections; its proliferation in the human body can possibly lead to infective endocarditis [53,54].

### 4.3. Antimicrobial Activity Screening of Each Natural Compound

Measurement of the diameter of the inhibition halos was performed, and, according to García-Díez et al. [55], only halos superior to 10 mm of diameter were defined as indicative of susceptibility of a given isolate to a specific compound (Table 4). Due to their antimicrobial activity and functional properties, essential oils, such as limonene, thymol, oleuropein, and carvacrol, have been the focus of several studies [15,17,18,19,20]. Solutions of 0.05% (*v/v*) carvacrol and limonene showed antimicrobial activity against 1.0% and 6.0% of the isolates, respectively (Table 4 and Table 5). Isolates that were inhibited by limonene were also inhibited by the emulsifier polysorbate 80, whereby this inhibition was not considered. Contrary to what was found in this study, Bunyan et al. [56] described carvacrol as an efficient antimicrobial agent against *Serratia* spp. with minimum inhibitory concentrations below 0.04% (*v/v*). Thielmann and Muranyi [57] reported minimum inhibitory concentrations of limonene for *Klebsiella* spp., *Leuconostoc* spp., and *Pseudomonas* spp. of 1.1%, 2.0%, and 0.8% (*v/v*), respectively. This indicates that in order to inhibit bacterial growth, much higher concentrations of limonene would have to be used. Curiously, the presence of these compounds in the disk appeared to improve the bacterial growth of some isolates. This occurred rarely, nonetheless, increased bacterial growth was observed 3–4 mm from the periphery of the disk. This result suggests that at some unknown concentration, polysorbate might induce bacterial proliferation. Similar findings were observed in the study by Nielsen et al. [58], where polysorbate 80 (0.1% *v/v*) added to the growth medium increased the growth rate of *S. aureus* while reducing the growth of *L. monocytogenes.* Nielsen et al. [58] also reported that the presence of polysorbate 80 reduced antimicrobial activity of both rifampicin and isoeugenol, indicating that polysorbate could be inhibiting the antibacterial activity of limonene, carvacrol, and olive leaf extract used in this present study.

Due to its nontoxic, biocompatible, safe-to-use, biodegradable, and antibacterial characteristics, chitosan has been suggested as a strong candidate for application in food products [59,60,61]. Chitosan is, however, only an efficient antimicrobial agent in acidic conditions, due to its solubility in low pH media [59]. In this study, all 3% (*w/v*) chitosan acidic solutions appeared to inhibit over 70% of the isolates (Table 4 and Table 5). The totality of *Pseudomonas* spp. and over 80% of isolates identified as belonging to the Enterobacterales order showed susceptibility to all four chitosan solutions tested. This agrees with the study of Cao et al. [62], where chitosan was also analyzed in the context of the shelf-life extension of molluscs. In that study, chitosan at 0.5% (*w/v*) was an effective antimicrobial against most isolates tested, including *Pseudomonas* spp. and Enterobacterales. While, in the present study, chitosan appeared to inhibit *Pseudomonas* spp. and Enterobacterales growth, the same isolates were also inhibited by the 3% (*v/v*) acetic acid used as control (Table 4 and Table 5). Because of this, it is not clear if the inhibition of bacterial growth was exclusively the result of the acetic acid in the solution. Therefore, the analysis of water-soluble chitosan might provide clearer information regarding the efficiency of chitosan, since the presence of acetic acid seems to overshadow the antibacterial activity of chitosan. Cao et al. [62] also observed inhibition of LAB isolates by a chitosan 0.1% (*w/v*) solution, with halos superior to 15 mm. In the present study, the chitosan acidic solution appeared to have a suboptimal antibacterial activity against LAB. It is speculated that this result is a consequence of a high resistance of LAB to acidic environments.

Propolis is a resinous mixture of beeswax and other resins collected by honeybees, usually of the *Apis melifera* species. It provides structural and protective action to the honeycomb [63]. While a strong antibacterial activity is commonly found as a characteristic of this compound [17], its antimicrobial activity is known to vary accordingly to the environmental characteristics from which it originates [63]. Ethanolic propolis extracts at 10% (*w/v*) inhibited the bacterial growth of some isolates, but this inhibition varied depending on the propolis used, being EPE2 slightly more efficient than EPE1. While inhibiting bacterial growth of some isolates, all of the susceptible isolates were LAB, which indicates that these solutions, while being efficient in controlling LAB proliferation, lack antibacterial activity against *Pseudomonas* and Enterobacterales. The results obtained are supported by the lack of bacterial inhibition by the ethanolic solution used as control, showing that antibacterial activity is a consequence of the propolis properties. Przybyłek and Karpiński [63] presented a compendium of studies of antibacterial activity of ethanolic propolis extracts against several bacteria with minimum inhibitory concentrations ranging from 5 μg/mL, against *Streptococcus sobrinius*, to over 2900 μg/mL, against *Salmonella* spp. While there is a considerable range of concentrations, the solution used in this study is considerably superior to those observed as efficient in the study of Przybyłek and Karpiński [63]. This might be the result of the known variability of propolis composition relative to the environmental conditions of where it is produced.

Citrox^®^ ProGarda solution managed to inhibit the growth of all isolates. This result presents Citrox^®^ as the product of interest, due to its broad-range activity capable of controlling bacterial proliferation in all isolates tested. Taking into account its composition based on a citrus extract, with phenolic compounds and organic acids as its active ingredients, microbial inhibition is due to both pH regulation and bioflavonoid activity. Tsiraki et al. [64,65,66] observed reductions in colony counts of *Bacillus cereus* [65], *Salmonella enterica* [65], and *Escherichia coli* O157:H7 [66] in Tzatziki, a traditional Greek yogurt-based salad, after the addition of Citrox^®^ ProGarda. Shelf-life extension of this traditional dish due to the impact of Citrox^®^ was also achieved [64]. Yehia et al. [67] also observed the total reduction of volatile basic nitrogen and methicillin-resistant *S. aureus* values in chicken meat after application of a Citrox^®^ solution; however, the solution used in this study had a different formulation. Since the purpose of this study was to collect information on natural compounds for their future use in the shelf-life extension of squid and shrimp skewers, of the above-mentioned solutions, only the application of a Citrox^®^ solution presented the recommended activity for the proposed objective.

### 4.4. Minimum Inhibitory and Bactericidal Concentrations of Citrox^®^

Regarding the minimum inhibitory concentration of Citrox^®^, when diluted to a final concentration of 1.69% (*v/v*) or higher, Citrox^®^ inhibited all the isolates tested. Citrox^®^ antibacterial activity decreased with increasing dilutions, inhibiting bacterial growth of only three isolates at the minimum concentration of 0.03%. Since the maximum recommended concentration for the spraying of foods with this product is 3% (*v/v*), the minimal inhibitory concentration observed was considered sufficient to achieve the proposed objective. To our knowledge, no studies about the MICs of Citrox^®^ ProGarda against the same species identified in this study are available in the literature. However, other formulations of Citrox^®^ are used for the disinfection of different surfaces, such as Citrox^®^ BC30 and Citrox^®^ MDC30 [68]. Different combinations of bioflavonoids with malic and citric acids cause the variation of its antibacterial activities; 2% (*v/v*) Citrox^®^ BC30 showed activity against *Clostridium difficile* while 8% (*v/v*) Citrox^®^ MDC30 was unable to inhibit the same pathogen [68].

All the suspensions where no growth was observed were plated on Müeller-Hinton Agar, and bacterial growth was observed in 97 out of the 100 isolates tested. This result indicates that Citrox^®^ at 1.69% mainly acted as a bacteriostatic compound, inhibiting bacterial growth but not destroying all bacterial cells. Considering that the shelf-life of the product is considerably short (3 to 4 days), and the purpose of any treatment is to extend it, the bacteriostatic properties of Citrox^®^ were deemed sufficient to the purpose intended, since the inhibition of bacterial growth and consequential spoilage for an additional 1 or 2 days would already considerably augment the freshness period and shelf-life of such perishable product.

## 5. Conclusions

In the present study, microbial characterization of squid and shrimp skewers was performed. Considerably high total viable counts were observed in the product. Counts on *Pseudomonas*-selective agar revealed that bacteria of this genus were the most numerous in the product. The high spoilage activity of *Pseudomonas* and its levels in this product confirm its fast perishability. *Listeria monocytogenes* and *E. coli* were not detected, which supports the idea that the processing of the product follows proper safety guidelines. One hundred selected bacteria were identified as Enterobacterales, *Pseudomonas*, or LAB, which shows a complete dominance of these bacterial groups in the microbiome of the product. Antimicrobial activity of limonene, carvacrol, olive leaf extract, propolis, Citrox^®^, and chitosan was tested against the isolated microorganisms. Solutions of limonene, carvacrol, and olive leaf extract lacked noteworthy antibacterial activity against the isolates tested. While all three chitosan solutions appeared to inhibit growth of a significant proportion of the isolates (>70%), the same activity was observed in the control solution. Ethanolic propolis extracts showed some efficiency in inhibiting LAB growth, but lacked antibacterial activity against the remaining isolates. Only Citrox^®^ inhibited the growth of all isolates, displaying bacteriostatic and bactericidal activity to 97% and 3% of the isolates, respectively, at concentrations higher or equal to 1.69% (*v/v*). While the need for further tests to prove this theory remains, the application of Citrox^®^ to seafood products presents itself as a potential shelf-life-extending strategy.

## Figures and Tables

**Table 1:** Microbial characterization of uncooked squid and shrimp skewers.

	log CFU/g					
	Standard Temperature			11 °C		
	Day 0	Day 2	Day 5	Day 0	Day 2	Day 5
*Listeria monocytogenes*	<1.00 ± 0.00	<1.00 ± 0.00	<1.00 ± 0.00	<1.00 ± 0.00	<1.00 ± 0.00	<1.00 ± 0.00
*Escherichia coli*	<1.00 ± 0.00	<1.00 ± 0.00	<1.00 ± 0.00	<1.00 ± 0.00	<1.00 ± 0.00	<1.00 ± 0.00
Total viable counts	5.49 ± 0.54	8.27 ± 0.25	9.46 ± 0.17	5.90 ± 0.51	8.64 ± 0.91	9.35 ± 0.11
Lactic Acid Bacteria	3.74 ± 0.15	6.13 ± 0.46	7.24 ± 0.10	4.65 ± <0.01	6.84 ± 0.18	7.69 ± 0.05
*Pseudomonas* spp.	6.21 ± 0.20	8.24 ± 0.31	9.20 ± 0.11	6.16 ± 0.18	8.42 ± 0.03	9.22 ± 0.12
Enterobacterales	4.71 ± 0.21	7.18 ± 0.8	8.31 ± 0.23	4.74 ± 0.01	7.42 ± 0.47	8.16 ± 0.02

**Table 2:** Number of isolates collected per culture medium.

	PCA	MRS	PAB	RAPID’ *Enterobacteriaceae*
Number of isolates	39	22	13	26

**Table 3:** Identification of 100 isolates by 16S rRNA sequencing.

Identification	Number of Isolates	16S rRNA Sequencing Similarity (%)
*Hafnia alvei*	6	99.40–99.90
*Klebsiella oxytoca*	2	99.90–100.00
*Latilactobacillus sakei*	5	99.90–100.00
*Lactococcus garvieae*	2	99.90–100.00
*Lactococcus piscium*	1	99.40–99.90
*Leuconostoc gelidum*	1	99.40–99.90
*Leuconostoc mesenteroides*	1	100.00
*Leuconostoc miyukkimchii*	12	99.10–99.90
*Morganella morganii*	3	99.80
*Obesumbacterium proteus*	1	99.90
*Pantoea brenneri*	1	98.50
*Pseudomonas endophytica*	1	99.70
*Pseudomonas fragi*	2	99.70
*Pseudomonas helleri*	3	99.80–100.00
*Pseudomonas helmanticensis*	1	99.80
*Pseudomonas marginalis*	1	100.00
*Pseudomonas orientalis*	1	99.80
*Pseudomonas paralactis*	6	99.80–100.00
*Pseudomonas psychrophila*	3	99.70–99.90
*Pseudomonas trivialis*	4	99.90
*Pseudomonas weihenstephanensis*	2	99.60–99.90
*Rahnella aquatilis*	4	99.60–99.90
*Rahnella inusitata*	10	99.80–99.90
*Serratia fonticola*	2	99.90
*Serratia liquefaciens*	20	99.40–99.90
*Serratia nematodiphila*	1	100.00
*Weissella fabalis*	3	97.10–98.10
*Weissella beninensis*	1	97.60

**Table 4:** Diameters of inhibition halos (mm) and susceptibility obtained for each compound against each identified isolate.

Car.	T80	OLE	Lim.	C. HMW1	C. HMW2	C. MMW	C. LMW	A.A.	EEP 1	EEP 2	ETOH	Citrox^®^	Identified Isolate
0 (R)	0 (R)	0 (R)	0 (R)	14 (S)	15 (S)	15 (S)	14 (S)	16 (S)	0 (R)	0 (R)	0 (R)	17 (S)	*Pseudomonas endophytica*
0 (R)	0 (R)	0 (R)	0 (R)	14 (S)	12 (S)	11 (S)	15 (S)	16 (S)	7 (R)	8 (R)	0 (R)	21 (S)	*Pseudomonas fragi*
0 (R)	0 (R)	0 (R)	0 (R)	14 (S)	12 (S)	11 (S)	15 (S)	16 (S)	7 (R)	8 (R)	0 (R)	21 (S)	*Pseudomonas fragi*
0 (R)	0 (R)	0 (R)	0 (R)	14 (S)	14 (S)	14 (S)	14 (S)	14 (S)	7 (R)	0 (R)	0 (R)	23 (S)	*Pseudomonas helleri*
0 (R)	0 (R)	0 (R)	0 (R)	21 (S)	24 (S)	27 (S)	27 (S)	24 (S)	9 (R)	8 (R)	0 (R)	31 (S)	*Pseudomonas helleri*
0 (R)	0 (R)	0 (R)	0 (R)	15 (S)	15 (S)	16 (S)	16 (S)	17 (S)	0 (R)	8 (R)	0 (R)	24 (S)	*Pseudomonas helleri*
0 (R)	0 (R)	0 (R)	0 (R)	14 (S)	15 (S)	14 (S)	16 (S)	15 (S)	9 (R)	0 (R)	0 (R)	24 (S)	*Pseudomonas helmanticensis*
0 (R)	0 (R)	0 (R)	0 (R)	16 (S)	16 (S)	14 (S)	16 (S)	20 (S)	0 (R)	9 (R)	0 (R)	31 (S)	*Pseudomonas marginalis*
0 (R)	0 (R)	0 (R)	0 (R)	14 (S)	15 (S)	14 (S)	15 (S)	14 (S)	0 (R)	0 (R)	0 (R)	25 (S)	*Pseudomonas orientalis*
0 (R)	0 (R)	0 (R)	0 (R)	16 (S)	17 (S)	17 (S)	15 (S)	15 (S)	7 (R)	8 (R)	0 (R)	22 (S)	*Pseudomonas paralactis*
0 (R)	0 (R)	0 (R)	0 (R)	15 (S)	12 (S)	16 (S)	16 (S)	17 (S)	8 (R)	7 (R)	0 (R)	23 (S)	*Pseudomonas paralactis*
0 (R)	0 (R)	0 (R)	0 (R)	14 (S)	15 (S)	13 (S)	15 (S)	13 (S)	0 (R)	7 (R)	0 (R)	25 (S)	*Pseudomonas paralactis*
0 (R)	0 (R)	0 (R)	0 (R)	20 (S)	21 (S)	21 (S)	22 (S)	23 (S)	0 (R)	8 (R)	0 (R)	30 (S)	*Pseudomonas paralactis*
0 (R)	0 (R)	0 (R)	0 (R)	17 (S)	20 (S)	17 (S)	20 (S)	20 (S)	9 (R)	0 (R)	0 (R)	30 (S)	*Pseudomonas paralactis*
0 (R)	0 (R)	0 (R)	0 (R)	14 (S)	15 (S)	14 (S)	16 (S)	18 (S)	8 (R)	8 (R)	7 (R)	30 (S)	*Pseudomonas paralactis*
0 (R)	0 (R)	0 (R)	0 (R)	18 (S)	15 (S)	20 (S)	15 (S)	16 (S)	7 (R)	8 (R)	0 (R)	22 (S)	*Pseudomonas psychrophila*
0 (R)	0 (R)	0 (R)	0 (R)	13 (S)	14 (S)	16 (S)	15 (S)	14 (S)	7 (R)	8 (R)	0 (R)	22 (S)	*Pseudomonas psychrophila*
0 (R)	0 (R)	0 (R)	0 (R)	12 (S)	17 (S)	15 (S)	18 (S)	16 (S)	9 (R)	8 (R)	0 (R)	24 (S)	*Pseudomonas psychrophila*
0 (R)	0 (R)	0 (R)	0 (R)	10 (S)	13 (S)	10 (S)	12 (S)	11 (S)	7 (R)	8 (R)	0 (R)	23 (S)	*Pseudomonas trivialis*
0 (R)	0 (R)	0 (R)	0 (R)	16 (S)	17 (S)	15 (S)	17 (S)	17 (S)	0 (R)	0 (R)	0 (R)	23 (S)	*Pseudomonas trivialis*
0 (R)	0 (R)	0 (R)	0 (R)	14 (S)	15 (S)	13 (S)	15 (S)	18 (S)	0 (R)	8 (R)	0 (R)	25 (S)	*Pseudomonas trivialis*
0 (R)	0 (R)	0 (R)	0 (R)	14 (S)	15 (S)	12 (S)	14 (S)	15 (S)	0 (R)	0 (R)	7 (R)	31 (S)	*Pseudomonas trivialis*
0 (R)	0 (R)	0 (R)	0 (R)	10 (S)	14 (S)	12 (S)	11 (S)	11 (S)	8 (R)	8 (R)	0 (R)	25 (S)	*Pseudomonas weihenstephanensis*
0 (R)	0 (R)	0 (R)	0 (R)	19 (S)	20 (S)	17 (S)	21 (S)	21 (S)	0 (R)	0 (R)	0 (R)	24 (S)	*Pseudomonas weihenstephanensis*
0 (R)	0 (R)	0 (R)	0 (R)	11 (S)	11 (S)	11 (S)	11 (S)	10 (S)	0 (R)	0 (R)	0 (R)	16 (S)	*Hafnia alvei*
0 (R)	0 (R)	0 (R)	0 (R)	11 (S)	12 (S)	10 (S)	10 (S)	11 (S)	0 (R)	8 (R)	0 (R)	21 (S)	*Hafnia alvei*
0 (R)	0 (R)	0 (R)	0 (R)	13 (S)	12 (S)	11 (S)	12 (S)	13(S)	0 (R)	0 (R)	0 (R)	15 (S)	*Hafnia alvei*
0 (R)	0 (R)	0 (R)	0 (R)	13 (S)	12 (S)	11 (S)	12 (S)	13 (S)	0 (R)	0 (R)	0 (R)	15 (S)	*Hafnia alvei*
0 (R)	0 (R)	0 (R)	0 (R)	10 (S)	10 (S)	9 (R)	10 (S)	12 (S)	8 (R)	0 (R)	7 (R)	20 (S)	*Hafnia alvei*
0 (R)	0 (R)	0 (R)	0 (R)	10 (S)	13 (S)	13 (S)	12 (S)	13 (S)	0 (R)	8 (R)	0 (R)	18 (S)	*Hafnia alvei*
0 (R)	0 (R)	0 (R)	0 (R)	11 (S)	12 (S)	10 (S)	12 (S)	13 (S)	0 (R)	7 (R)	0 (R)	19 (S)	*Klebsiella oxytoca*
0 (R)	0 (R)	0 (R)	0 (R)	12 (S)	14 (S)	13 (S)	14 (S)	15 (S)	0 (R)	8 (R)	0 (R)	16 (S)	*Klebsiella oxytoca*
0 (R)	0 (R)	0 (R)	0 (R)	15 (S)	15 (S)	13 (S)	15 (S)	16 (S)	8 (R)	0 (R)	0 (R)	23 (S)	*Morganella morganii*
0 (R)	0 (R)	0 (R)	0 (R)	16 (S)	14 (S)	14 (S)	14 (S)	15 (S)	0 (R)	7 (R)	0 (R)	25 (S)	*Morganella morganii*
0 (R)	0 (R)	0 (R)	0 (R)	10 (S)	10 (S)	10 (S)	10 (S)	10 (S)	8 (R)	8 (R)	0 (R)	22 (S)	*Morganella morganii*
0 (R)	0 (R)	0 (R)	0 (R)	8 (R)	8 (R)	8 (R)	9 (R)	7 (R)	8 (R)	7 (R)	0 (R)	14 (S)	*Obesumbacterium proteus*
0 (R)	0 (R)	0 (R)	0 (R)	11 (S)	10 (S)	11 (S)	11 (S)	12 (S)	7 (R)	7 (R)	0 (R)	15 (S)	*Pantoea brenneri*
0 (R)	0 (R)	0 (R)	0 (R)	17 (S)	16 (S)	17 (S)	16 (S)	18 (S)	8 (R)	9 (R)	0 (R)	23 (S)	*Rahnella aquatilis*
0 (R)	0 (R)	0 (R)	0 (R)	17 (S)	18 (S)	16 (S)	17 (S)	18 (S)	0 (R)	0 (R)	0 (R)	21 (S)	*Rahnella aquatilis*
0 (R)	0 (R)	0 (R)	0 (R)	16 (S)	18 (S)	15 (S)	16 (S)	15 (S)	0 (R)	8 (R)	0 (R)	16 (S)	*Rahnella aquatilis*
0 (R)	0 (R)	0 (R)	0 (R)	14 (S)	15 (S)	14 (S)	17 (S)	18 (S)	0 (R)	7 (R)	0 (R)	23 (S)	*Rahnella aquatilis*
0 (R)	0 (R)	0 (R)	0 (R)	0 (R)	0 (R)	0 (R)	0 (R)	0 (R)	0 (R)	0 (R)	0 (R)	15 (S)	*Rahnella inusitata*
0 (R)	0 (R)	0 (R)	0 (R)	13 (S)	13 (S)	10 (S)	10 (S)	11 (S)	7 (R)	9 (R)	0 (R)	19 (S)	*Rahnella inusitata*
0 (R)	0 (R)	0 (R)	0 (R)	12 (S)	12 (S)	13 (S)	11 (S)	11 (S)	8 (R)	7 (R)	0 (R)	18 (S)	*Rahnella inusitata*
0 (R)	0 (R)	0 (R)	0 (R)	12 (S)	12 (S)	12 (S)	15 (S)	13 (S)	7 (R)	0 (R)	0 (R)	17 (S)	*Rahnella inusitata*
0 (R)	0 (R)	0 (R)	0 (R)	14 (S)	13 (S)	11 (S)	13 (S)	13 (S)	0 (R)	7 (R)	0 (R)	33 (S)	*Rahnella inusitata*
0 (R)	0 (R)	0 (R)	0 (R)	22 (S)	22 (S)	24 (S)	23 (S)	23 (S)	9 (R)	7 (R)	0 (R)	20 (S)	*Rahnella inusitata*
0 (R)	0 (R)	0 (R)	0 (R)	11 (S)	12 (S)	11 (S)	12 (S)	13 (S)	0 (R)	8 (R)	7 (R)	19 (S)	*Rahnella inusitata*
0 (R)	0 (R)	0 (R)	0 (R)	16 (S)	17 (S)	16 (S)	16 (S)	17 (S)	0 (R)	0 (R)	0 (R)	25 (S)	*Rahnella inusitata*
0 (R)	0 (R)	0 (R)	0 (R)	17 (S)	19 (S)	19 (S)	20 (S)	19 (S)	9 (R)	0 (R)	0 (R)	22 (S)	*Rahnella inusitata*
0 (R)	0 (R)	0 (R)	0 (R)	15 (S)	15 (S)	14 (S)	14 (S)	16 (S)	0 (R)	7 (R)	0 (R)	22 (S)	*Rahnella inusitata*
0 (R)	0 (R)	0 (R)	0 (R)	11 (S)	14 (S)	12 (S)	14 (S)	14 (S)	7 (R)	7 (R)	0 (R)	18 (S)	*Serratia fonticola*
0 (R)	0 (R)	0 (R)	0 (R)	12 (S)	14 (S)	13 (S)	12 (S)	14 (S)	0 (R)	8 (R)	0 (R)	19 (S)	*Serratia fonticola*
8 (R)	10 (S)	0 (R)	9 (R)	15 (S)	16 (S)	14 (S)	17 (S)	15 (S)	0 (R)	0 (R)	0 (R)	19 (S)	*Serratia liquefaciens*
8 (R)	11 (S)	0 (R)	10 (S)	11 (S)	11 (S)	10 (S)	9 (R)	10 (S)	7 (R)	8 (R)	8 (R)	17 (S)	*Serratia liquefaciens*
0 (R)	0 (R)	0 (R)	0 (R)	11 (S)	8 (R)	10 (S)	8 (R)	10 (S)	7 (R)	0 (R)	0 (R)	19 (S)	*Serratia liquefaciens*
7 (R)	9 (R)	0 (R)	9 (R)	14 (S)	15 (S)	12 (S)	14 (S)	15 (S)	8 (R)	8 (R)	13 (S)	18 (S)	*Serratia liquefaciens*
0 (R)	0 (R)	0 (R)	0 (R)	8 (R)	9 (R)	7 (R)	9 (R)	8 (R)	0 (R)	7 (R)	0 (R)	18 (S)	*Serratia liquefaciens*
0 (R)	0 (R)	0 (R)	0 (R)	12 (S)	12 (S)	10 (S)	12 (S)	15 (S)	8 (R)	0 (R)	0 (R)	16 (S)	*Serratia liquefaciens*
8 (R)	8 (R)	0 (R)	9 (R)	12 (S)	15 (S)	13 (S)	13 (S)	14 (S)	8 (R)	7 (R)	0 (R)	12 (S)	*Serratia liquefaciens*
9 (R)	10 (S)	0 (R)	10 (S)	15 (S)	15 (S)	15 (S)	13 (S)	15 (S)	7 (R)	8 (R)	0 (R)	18 (S)	*Serratia liquefaciens*
9 (R)	9 (R)	0 (R)	10 (S)	15 (S)	13 (S)	14 (S)	14 (S)	14 (S)	8 (R)	7 (R)	0 (R)	19 (S)	*Serratia liquefaciens*
9 (R)	11 (S)	0 (R)	10 (S)	10 (S)	8 (R)	10 (S)	10 (S)	7 (R)	8 (R)	7 (R)	0 (R)	18 (S)	*Serratia liquefaciens*
8 (R)	9 (R)	0 (R)	9 (R)	9 (R)	0 (R)	8 (R)	8 (R)	7 (R)	0 (R)	0 (R)	0 (R)	16 (S)	*Serratia liquefaciens*
9 (R)	9 (R)	0 (R)	9 (R)	12 (S)	13 (S)	12 (S)	12 (S)	12 (S)	7 (R)	7 (R)	0 (R)	20 (S)	*Serratia liquefaciens*
0 (R)	0 (R)	0 (R)	0 (R)	16 (S)	14 (S)	12 (S)	18 (S)	16 (S)	0 (R)	8 (R)	0 (R)	19 (S)	*Serratia liquefaciens*
8 (R)	8 (R)	0 (R)	8 (R)	0 (R)	12 (S)	12 (S)	12 (S)	12 (S)	0 (R)	8 (R)	0 (R)	17 (S)	*Serratia liquefaciens*
0 (R)	0 (R)	0 (R)	0 (R)	20 (S)	20 (S)	20 (S)	20 (S)	24 (S)	0 (R)	0 (R)	7 (R)	20 (S)	*Serratia liquefaciens*
9 (R)	10 (S)	0 (R)	10 (S)	24 (S)	22 (S)	16 (S)	20 (S)	20 (S)	0 (R)	0 (R)	0 (R)	20 (S)	*Serratia liquefaciens*
0 (R)	0 (R)	0 (R)	0 (R)	21 (S)	19 (S)	19 (S)	19 (S)	24 (S)	9 (R)	8 (R)	0 (R)	22 (S)	*Serratia liquefaciens*
0 (R)	0 (R)	0 (R)	0 (R)	14 (S)	14 (S)	13 (S)	15 (S)	13 (S)	0 (R)	7 (R)	0 (R)	19 (S)	*Serratia liquefaciens*
0 (R)	0 (R)	0 (R)	0 (R)	11 (S)	13 (S)	15 (S)	14 (S)	16 (S)	7 (R)	0 (R)	0 (R)	20 (S)	*Serratia liquefaciens*
0 (R)	0 (R)	0 (R)	0 (R)	0 (R)	0 (R)	0 (R)	0 (R)	0 (R)	0 (R)	0 (R)	0 (R)	18 (S)	*Serratia liquefaciens*
7 (R)	9 (R)	0 (R)	7 (R)	8 (R)	13 (S)	11 (S)	12 (S)	12 (S)	0 (R)	0 (R)	0 (R)	17 (S)	*Serratia nematodiphila*
0 (R)	0 (R)	0 (R)	0 (R)	0 (R)	0 (R)	0 (R)	0 (R)	0 (R)	0 (R)	0 (R)	0 (R)	17 (S)	*Latilactobacillus sakei*
0 (R)	0 (R)	0 (R)	0 (R)	0 (R)	0 (R)	0 (R)	0 (R)	0 (R)	0 (R)	0 (R)	0 (R)	15 (S)	*Latilactobacillus sakei*
0 (R)	0 (R)	0 (R)	0 (R)	0 (R)	0 (R)	0 (R)	0 (R)	0 (R)	0 (R)	11 (S)	0 (R)	19 (S)	*Latilactobacillus sakei*
0 (R)	0 (R)	0 (R)	0 (R)	0 (R)	0 (R)	0 (R)	0 (R)	10 (S)	12 (S)	12 (S)	0 (R)	30 (S)	*Latilactobacillus sakei*
0 (R)	0 (R)	0 (R)	0 (R)	0 (R)	0 (R)	0 (R)	0 (R)	0 (R)	0 (R)	0 (R)	0 (R)	13 (S)	*Latilactobacillus sakei*
0 (R)	0 (R)	0 (R)	0 (R)	0 (R)	0 (R)	0 (R)	0 (R)	10 (S)	11 (S)	10 (S)	0 (R)	30 (S)	*Lactococcus garvieae*
0 (R)	0 (R)	0 (R)	0 (R)	0 (R)	0 (R)	0 (R)	0 (R)	0 (R)	12 (S)	10 (S)	0 (R)	32 (S)	*Lactococcus garvieae*
0 (R)	0 (R)	0 (R)	0 (R)	18 (S)	17 (S)	15 (S)	16 (S)	18 (S)	7 (R)	8 (R)	0 (R)	21 (S)	*Lactococcus piscium*
0 (R)	0 (R)	0 (R)	0 (R)	0 (R)	0 (R)	0 (R)	0 (R)	0 (R)	0 (R)	10 (S)	0 (R)	15 (S)	*Leuconostoc gelidum*
0 (R)	0 (R)	0 (R)	0 (R)	0 (R)	0 (R)	0 (R)	0 (R)	0 (R)	0 (R)	11 (S)	0 (R)	21 (S)	*Leuconostoc mesenteroides*
0 (R)	0 (R)	0 (R)	0 (R)	0 (R)	0 (R)	0 (R)	0 (R)	12 (S)	18 (S)	12 (S)	0 (R)	40 (S)	*Leuconostoc miyukkimchii*
12 (S)	10 (S)	7 (R)	10 (S)	8 (R)	8 (R)	8 (R)	8 (R)	0 (R)	7 (R)	8 (R)	0 (R)	26 (S)	*Leuconostoc miyukkimchii*
0 (R)	0 (R)	0 (R)	0 (R)	0 (R)	0 (R)	0 (R)	0 (R)	10 (S)	12 (S)	12 (S)	0 (R)	30 (S)	*Leuconostoc miyukkimchii*
0 (R)	0 (R)	0 (R)	0 (R)	0 (R)	0 (R)	0 (R)	0 (R)	8 (R)	12 (S)	15 (S)	0 (R)	28 (S)	*Leuconostoc miyukkimchii*
0 (R)	0 (R)	0 (R)	0 (R)	0 (R)	0 (R)	0 (R)	0 (R)	10 (S)	15 (S)	12 (S)	0 (R)	32 (S)	*Leuconostoc miyukkimchii*
0 (R)	0 (R)	0 (R)	0 (R)	0 (R)	0 (R)	0 (R)	0 (R)	10 (S)	13 (S)	10 (S)	0 (R)	30 (S)	*Leuconostoc miyukkimchii*
0 (R)	0 (R)	0 (R)	0 (R)	11 (S)	11 (S)	10 (S)	11 (S)	11 (S)	8 (R)	8 (R)	7 (R)	20 (S)	*Leuconostoc miyukkimchii*
0 (R)	0 (R)	0 (R)	0 (R)	0 (R)	0 (R)	0 (R)	0 (R)	0 (R)	10 (S)	12 (S)	0 (R)	19 (S)	*Leuconostoc miyukkimchii*
0 (R)	0 (R)	0 (R)	0 (R)	0 (R)	0 (R)	0 (R)	0 (R)	10 (S)	14 (S)	12 (S)	0 (R)	34 (S)	*Leuconostoc miyukkimchii*
0 (R)	0 (R)	0 (R)	0 (R)	0 (R)	0 (R)	0 (R)	0 (R)	13 (S)	16 (S)	14 (S)	0 (R)	38 (S)	*Leuconostoc miyukkimchii*
0 (R)	0 (R)	0 (R)	0 (R)	0 (R)	0 (R)	0 (R)	0 (R)	10 (S)	13 (S)	14 (S)	0 (R)	40 (S)	*Leuconostoc miyukkimchii*
0 (R)	0 (R)	0 (R)	0 (R)	15 (S)	15 (S)	13 (S)	15 (S)	15 (S)	8 (R)	8 (R)	0 (R)	25 (S)	*Leuconostoc miyukkimchii*
0 (R)	0 (R)	0 (R)	0 (R)	0 (R)	0 (R)	0 (R)	0 (R)	0 (R)	0 (R)	10 (S)	0 (R)	28 (S)	*Weissella fabalis*
0 (R)	0 (R)	0 (R)	0 (R)	0 (R)	0 (R)	0 (R)	0 (R)	0 (R)	0 (R)	10 (S)	0 (R)	26 (S)	*Weissella beninensis*
0 (R)	0 (R)	0 (R)	0 (R)	0 (R)	0 (R)	0 (R)	0 (R)	0 (R)	0 (R)	10 (S)	0 (R)	24 (S)	*Weissella fabalis*
0 (R)	0 (R)	0 (R)	0 (R)	0 (R)	0 (R)	0 (R)	0 (R)	0 (R)	0 (R)	10 (S)	0 (R)	20 (S)	*Weissella fabalis*

Legend: Car. (carvacrol), T80 (polysorbate), Lim. (limonene), C. HMW (chitosan high molecular weight), MMW (medium molecular weight), LMW (low molecular weight), A.A. (acetic acid), EEP (ethanolic extract of propolis), ETOH (ethanol), R (resistant), S (sensitive).

**Table 5:** Screening of antimicrobial activity of 10 natural compounds and respective controls.

Compounds	% Sensitivity *Pseudomonas*	% Sensitivity Enterobacterales	% Sensitivity LAB	% Sensitivity Total
0.05% (*v/v*) Carvacrol	0.0	0.0	3.8	1.0
Polysorbate 80	0.0	10.0	3.8	6.0
1.5% (*w/v*) Olive leaf extract	0.0	0.0	0.0	0.0
0.05% (*v/v*) Limonene	0.0	10.0	3.8	6.0
3% (*v/v*) Chitosan High Weight 1	100.0	86.0	11.5	70.0
3% (*v/v*) Chitosan High Weight 2	100.0	86.0	11.5	70.0
3% (*v/v*) Chitosan Medium Weight	100.0	88.0	11.5	71.0
3% (*v/v*) Chitosan Low Weight	100.0	86.0	11.5	70.0
3% (*v/v*) Acetic Acid	100.0	88.0	46.2	80.0
10% (*w/v*) EPE 1	0.0	0.0	46.2	12.0
10% (*w/v*) EPE 2	0.0	0.0	73.1	19.0
70% (*v/v*) Ethanol	0.0	2.0	0.0	1.0
50% (*v/v*) Citrox^®^	100.0	100.0	100.0	100.0

Legend: EPE—ethanolic propolis extract.

**Table 6:** Inhibitory effect of Citrox^®^ solutions at different concentrations.

% (*v/v*) Citrox^®^	% Sensitivity
50.00	100
25.00	100
12.50	100
6.75	100
3.38	100
1.69	100
0.84	53
0.42	17
0.21	13
0.11	8
0.06	3
0.03	3

## Data Availability

The datasets generated for this study are available on request to the corresponding author.
